# Applicability of visceral adiposity index in predicting metabolic syndrome in adults with obstructive sleep apnea: a cross-sectional study

**DOI:** 10.1186/s12890-016-0198-0

**Published:** 2016-03-01

**Authors:** Gong-Ping Chen, Jia-Chao Qi, Bi-Ying Wang, Xin Lin, Xiao-Bin Zhang, Jian-Ming Zhao, Xiao Fang Chen, Ting Lin, Dong-Dong Chen, Qi-Chang Lin

**Affiliations:** Department of Respiratory Medicine, Fujian Provincial Sleep-disordered Breathing Clinic Center; Laboratory of Respiratory Disease of the Fujian Medical University, the First Affiliated Hospital of Fujian Medical University, No. 20, Chazhong Road, Taijiang District, Fuzhou, 350005 Fujian Province People’s Republic of China; Department of Respiratory Medicine, Zhongshan Hospital, Xiamen University, Teaching Hospital of Fujian Medical University, No. 201, Hubin Nan Road, Siming District, Xiamen, 361004 Fujian Province People’s Republic of China

**Keywords:** Obstructive sleep apnea, Visceral adiposity, Visceral adiposity index, Metabolic syndrome, Metabolic score, Receiver operating characteristic

## Abstract

**Background:**

Obstructive sleep apnea (OSA) is severely affected by visceral adiposity (VA) that correlates to another disorder—metabolic syndrome (MetS). However, little is known concerning the relation of visceral adiposity index (VAI)—a novel and simple indicator of VA, with OSA and MetS. The objective of the study was to analyze the association of VAI with both disorders and applicability to identify OSA patients at risk of MetS.

**Methods:**

Consecutive individuals undergoing polysomnography and biochemical tests were enrolled, and differences in all subjects grouped by apnea-hypopnea index (AHI) were analyzed. Spearman correlation was performed for assessing the relationship between VAI, OSA-related indices and metabolic score—total number of the positive diagnostic criteria of MetS. Receiver operating characteristic (ROC) curve was conducted to obtain a cut-off value of VAI for predicting incident MetS by sex. Then, the risk of MetS in OSA patients according to the cut-offs was attained by logistic regression.

**Results:**

A total of 411 individuals were enrolled. Of whom, 361 subjects were diagnosed OSA (mild in 67 patients, moderate in 89 and severe in 205, respectively). A significant increasing trend based on AHI was observed in the variables of blood pressure, triglycerides, fasting glucose, incident MetS, metabolic score and VAI (all *p* < 0.05). Irrespective of gender, VAI was all significantly correlated with PSG characteristics as AHI, mean nocturnal oxygen saturation, the lowest oxygen saturation, metabolic score(all *p* < 0.05). A VAI of 2.282, 2.105, 2.511 (for all subjects, males and females, separately) were calculated to determine the occurrence of MetS. According to the cut-offs, OSA patients tended to suffer from greater risk in MetS (odds ratio [OR] = 10.237, *p* = 0.000; OR = 13.556, *p* = 0.000; OR = 21.458, *p* = 0.000).

**Conclusions:**

The present study suggested that VAI was significantly associated with MetS and OSA. As a simple and alternative approach obtained in everyday practice, it may offer a powerful tool to identify patients with OSA at risk of MetS.

## Background

Obstructive sleep apnea (OSA) is a highly prevalent disorder with 23.4–49.7 % of the general population. It refers to repetitive events of partial or complete upper airway occlusion during sleep, leading to recurrent oxyhemoglobin desaturations [1]. OSA is mainly determined by visceral adiposity that correlates to another disorder—metabolic syndrome (MetS) [2, 3]. MetS, with a prevalence of 22.8 % of men and 22.6 % of women in general population, is recognized as a constellation of visceral obesity, glucose intolerance, dyslipidemia and hypertension [4]. It predisposed patients to the development of cardiovascular diseases (CVD) and diabetes [5]. To our knowledge, there is growing amount of evidence on the relationship of OSA with MetS [6, 7].

Visceral adiposity, accurately estimated by computed tomography (CT), has been confirmed to be an important contributor for not only MetS but also [2, 3, 8] OSA. Meanwhile, regardless of at a similar body mass index (BMI) or waist circumference (WC), Chinese individuals are prone to have a greater amount of visceral adipose tissue than Europeans [9]. Thus, visceral adiposity index (VAI) as a reliable surrogate gender-specific indicator of adipose tissue function and distribution, which comprises simple anthropometric (BMI and WC) and metabolic (triglycerides (TG) and high density lipoprotein-cholesterol (HDL-C)) markers, is implicated in insulin sensitivity, MetS and cardio-metabolic diseases [10–12].

To date, little information is available regarding the relation between VAI and OSA. Recently, one study demonstrated the link between MetS and VAI in OSA patients. It was reported that VAI progressively increased with insulin resistance (IR) and metabolic score, unfortunately, it did not predict sleep apnea severity, suggesting that VAI was a good marker of MetS, but not of OSA [13]. On the other hand, the applicability of VAI in early determining cardio-metabolic risk, particularly in those subjects who did not have overt MetS, has been described in several studies [14–16]. VAI was superior to other metabolic markers in discriminating metabolism abnormalities, adipokine secretion and adipose tissue distribution [17, 18].

The aim of the current study was to explore whether VAI was closely associated with both OSA and MetS, and to further test the applicability of VAI in predicting MetS in OSA patients, improving the management of the complication of OSA.

## Method

### Study population

From January 2011 to December 2014, a total of 411 individuals, who were on admission to our sleep center with the complaints suggestive of nocturnal hypopnea, were retrospectively analyzed. These subjects completed a detailed questionnaire containing histories of current smoking and illness, medical treatment including medications for hypertension and hypertriglyceridemia, hyperglycemia prior to the study, and they underwent an overnight polysomnography (PSG). According to self-reported medical histories, patients who were excluded met the criterion below: 1. chronic obstructive pulmonary disease; 2. Asthma; 3. Cerebrovascular disease; 4. Congestive heart failure; 5. Symptomatic ischemic heart disease; 6. Chronic renal failure; 7. Rheumatologic diseases and Hypothyroidism. Those previously diagnosed or treated for OSA were also excluded. The research was approved by the Ethics Committee of the First Affiliated Hospital of Fujian Medical University. All patients provided written informed consent to participate in this study.

### Anthropometric and clinical assessment

The details of demographic parameters including age, gender, WC, height, weight and blood pressure (BP) were obtained. Weight and height were measured, with subjects in light clothing without shoes in a standing position while shoulders were in a normal alignment. And BMI was calculated based on the equation—weight is divided by the square of height (kg/m^2^). WC was measured at the umbilical level. After a 5-min interval, two measurements of BP were taken by well-trained physicians, on the right arm, using a standardized mercury sphygmomanometer in the sitting position; Then, the average was described in the data analysis. Venous Blood was drawn after 12-h fasting period for measurement of fasting glucose (FG), HDL and TG using the Modular P800 auto-analyze (Roche, Tokyo, Japan). VAI, a sex-specific index based on WC, BMI, TG and HDL-C, was obtained using the following equations [10]: Males: VAI = (WC/(39.68 + (1.886BMI)))*(TG/1.03)* (1.31/HDL). Females: VAI = (WC/(36.58 + (1.896BMI)))* (TG/0.81)* (1.52/ HDL).

### Definition of metabolic syndrome

Patients were diagnosed MetS based on the NCEP ATP III modified criteria [7] which describes a combination of at least three components of the five abnormalities: 1) waist circumference ≥90 cm for men and ≥80 cm for women of Asian; 2) a serum triglycerides ≥1.7 mmol/L or the use of medication for dyslipidemia; 3) HDL < 40 mg/dL for men and <50 mg/dL for women or the use of medication for low HDL; 4) a recorded arterial BP ≥130 or ≥85 mmHg for systolic and diastolic BP separately according to the average of two consecutive measurements, or the use of antihypertensive medication; 5) FG level ≥ 5.6 mmol/L or the use of diabetes medication. The metabolic score was determined as the sum of the positive MetS diagnostic criteria for each participant.

### Polysomnography evaluation

A full-night PSG (P Series Sleep System; Compumedics; Melbourne, Australia) monitoring recorded parameters as follows: electroencephalogram, oculogram, chin and bilateral anterior tibials electromyogram, electrocardiogram, nasal airflow, chest and abdominal movement, pulse oxygen saturation, snoring, and body position. All tracings were scored manually according to the guideline of the American Academy of Sleep Medicine (AASM) in 2007 [19]. Apnea was defined as a complete cessation (≥90 %) from baseline in airflow of ≥10s and hypopnea as a drop (≥30 %) in airflow of ≥10s accompanied by oxygen desaturation ≥4 %. Apnea-hypopnea index (AHI) was expressed as a total number of episodes of obstructive apneas plus hypopneas per hour during sleep. Oxygen desaturation index (ODI) was attained from the frequency of desaturations of ≥4 % per hour. The percentage of sleep duration with SpO_2_ < 90 % (TS90 %), and lowest O_2_ saturation (LaSO_2_), mean oxygen saturation (mean SpO_2_) were also recorded. Subjects were classified into four groups according to AHI. OSA was defined as absent, mild, moderate and severe when AHI was < 5/h, 5–14.9/h, 15–29.9/h, and > 30/h, respectively.

### Statistics analysis

All analyses were conducted using SPSS version 20.0 (SPSS Inc., Armonk, NY, USA). All variables were assessed for normality by Kolmogorov–Smirnov test. Skewed continuous variables presented as the median and interquartile range (IQR) and were analyzed by Kruskal–Wallis H. Normally distributed data presented as mean ± SD were analyzed by Student’s *t* test or one-way ANOVA for comparison. Categorical variables were presented as number (percentage), applying the chi-square test or Fisher’s exact test when compared. Spearman correlation was performed for assessing the relationship between VAI, metabolic score and OSA-related hypoxemia indices. Variables that did not have a normal distribution were log-transformed. We conducted receiver operating characteristic curve (ROC) and calculated the area below the curves with a 95 % confidence interval. A cut-off value of VAI by sex for predicting the presence of MetS was attained. Afterwards, a binary logistic regression was performed to analyze the risk of incident MetS in OSA patients based on VAI’s cut-off points. A two-tailed value of *p* < 0.05 was thought to be significant.

## Results

A total of 411 individuals, including 312 males and 99 females, were enrolled in the study. Their BMI were 26.49 ± 3.03 kg/m2, ages were 48.80 ± 13.62 years. Of whom, 361 subjects were found AHI ≥ 5/h, with mild in 67 patients, moderate in 89 and severe in 205, respectively. The demographic and polysomnographic parameters of all subjects according to AHI are detailed in Table 1. No differences could be found for age, current smoking or medical treatment, whereas gender was significantly different among groups. WC, BMI, AHI, ODI and TS90 % progressively increased across AHI categories, while mean SpO_2_, LaSO_2_ decreased significantly (all *p* < 0.001).

**Table 1:** The anthropometric and polysomnographic parameters of all patients according to the severity of OSA

	AHI (0-5)	AHI (5-15)	AHI (15-30)	AHI (30-)	*P*
Subjects	50	67	89	205	
Age, years	44.36 ± 16.18	49.70 ± 11.64	49.72 ± 12.98	49.45 ± 13.62	0.105
Male sex, number (%)	30 (60 %)	41 (61.19 %)*****	69(77.52 %)	172(83.9 %)***, *****	0.000
Current smoking, number (%)	6(12 %)	12(17.91 %)	19(21.35 %)	44(21.46 %)	0.445
Antilipimic agents, number (%)	2(4 %)	3(4.48 %)	4(4.49 %)	14(6.83 %)	0.772
Antidiabetic agents, number (%)	1(2 %)	4(5.97 %)	6(6.74 %)	16(7.80 %)	0.522
Antihypertensive agents, number (%)	8(16 %)	12(17.91 %)	27(30.34 %)	56(27.32 %)	0.108
Waist circumference (cm)	88.70 ± 10.35**, ***	90.43 ± 8.79****, *****	94.58 ± 8.43**, ****, ******	98.26 ± 10.27***, *****, ******	0.000
BMI (kg/m)^2^	23.94 ± 3.03**, ***	24.98 ± 3.25*****	25.94 ± 3.69**, ******	27.84 ± 5.93***, *****, ******	0.000
AHI	2.14(0.80-4.25)*, **, ***	9.30(7.20-12.30)*, ****, *****	21.30(18.25-24.25)**, ****, ******	52.40(40.95-65.45)***, *****, ******	0.000
Mean SpO2 (%)	96.50 (95.00-97.00)**, ***	96.00(95.00-96.00)*****	95.00(94.00-96.00)**, ******	92.00(88.00-94.00)***, *****, ******	0.000
LaSO2 (%)	90.00(84.75-92.00)**, ***	86.00(80.00-88.00)****, *****	79.00(75.00-85.50)**, ****, ******	67.00(57.00-78.00)***, *****, ******	0.000
TS90% (%)	0.00(0.00-0.36)**, ***	0.18(0.02-0.78)*****	0.98(0.10-2.57)**, ******	7.60(2.71-17.93)***, *****, ******	0.000
ODI	1.50(0.50-3.40)**, ***	6.30(3.40-9.40)****, *****	15.54(9.58-19.95)**, ****, ******	46.20(31.60-62.40)***, *****, ******	0.000

Normally distributed data (age, waist circumference, BMI) were expressed as mean ± *SD* skewed data (including, AHI, ODI, TS90 %, LaSO2, mean SpO2) were presented as median (interquartile range). Categorical variables were expressed as number (percentage), *OSA* obstructive sleep apnea, *BMI* body mass index, *AHI* apnea-hypopnea index; mean SpO2: mean nocturnal oxygen saturation, *LaSO2* lowest O2 saturation, *TS90%* the percentage of sleep duration with SpO2 < 90 %, *ODI* oxygen desaturation index; *: if *P* < 0.05 between group 0 [AHI (0-5)] and group 1 [AHI(5-15)]; **: if *P* < 0.05 between group 0 [AHI (0-5)] and group 2 [AHI(15-30)]; ***: if *P* < 0.05 between group 0 [AHI (0-5)] and group 3[AHI(30-)]; ****: if *P* < 0.05 between group 1 and group 2; *****: if *P* < 0.05 between group 1 and group 3; ******: if *P* < 0.05 between group 2 and group 3

The metabolic characteristics of the participants are shown in Table 2. A significant increasing trend based on AHI was observed in the variables of SBP, TG, FG, uric acid (UA), incident MetS, metabolic score and VAI (all *p* < 0.05). Conversely, a significantly negative association of AHI with HDL-C was presented (*p* = 0.000).

**Table 2:** The metabolic and biochemical characteristics of all patients according to the severity of OSA

	AHI (0-5)	AHI (5-15)	AHI (15-30)	AHI (30-)	*P*
SBP (mmHg)	120.00(110.00-130.00)***	120.00(117.00-138.00)	128.00 (120.00-140.00)	130.00 (120.00-140.00)***	0.005
DBP (mmHg)	80.00(71.50-80.00)***	78.00(70.00-80.00)*****	80.00(76.00-84.50)	80.00 (77.00-88.00)***, *****	0.005
HDL-C (mmol/L)	1.28 (1.01-1.54)***	1.19(1.03-1.47)*****	1.16 (0.93-1.37)	1.04(0.91-1.26)***, *****	0.000
Fasting glucose (mmol/L)	5.02(4.54-5.54)***	5.02(4.73-5.82)	5.16(4.76-5.68)	5.33(4.91-6.00)***	0.008
Triglycerides (mmol/L)	1.61 ± 1.07***	1.66 ± 0.89*****	1.76 ± 1.14******	2.32 ± 1.94***, *****, ******	0.001
Uric acid (mmol/L)	350.66 ± 97.10***	336.24 ± 103.99****, *****	378.12 ± 96.41****, ******	407.98 ± 103.36***, *****, ******	0.000
Hypertension, number (%)	30(60 %)***	40(59.7 %)	66(74.16 %)	157(76.59 %)***	0.014
Hypertriglyceride mia, number (%)	16(32 %)***	25(37.31 %)*****	34(38.20 %)******	114(56.10 %)*****, ******	0.001
Hyperglycemia, number (%)	11(22 %)	21(31.34 %)	28(31.46 %)	76(37.07 %)	0.188
Low HDL-C, number (%)	15(30 %)***	25(37.31 %)	36(40.45 %)	105(51.22 %)***	0.017
Central obesity, number (%)	31(62 %)**, ***	54(80.60 %)	73(82.02 %)**	175(85.37 %)***	0.003
Metabolic syndrome, number (%)	16(32 %)***	34(50.74 %)	48(53.93 %)	140(68.29 %)***	0.000
Metabolic score	2.00 (1.00-3.00)***	3.00(2.00-3.00)*****	3.00(2.00-4.00)	3.00(2.00-4.00)*****	0.000
VAI (All)	1.46 (0.99-2.75)***	1.90(1.25-2.89)*****	1.93(1.25-3.01)******	2.53(1.70-3.79)*****, ******	0.000
VAI (Male)	1.62(1.41-4.11)	2.04(1.50-2.95)	1.94(1.26-2.93)	2.52(1.61-3.74)	0.027
VAI (Female)	1.19(0.71-2.35)	1.52(0.93-2.83)	1.80(0.94-3.74)	2.54(1.82-4.32)	0.001

Normally distributed data (triglycerides, uric acid) were expressed as mean ± *SD* skewed data (including SBP/DBP, HDL-C, fasting glucose, metabolic score, VAI) were presented as median (interquartile range). Categorical variables were expressed as number (percentage). OSA: obstructive sleep apnea, *SBP/DBP* systolic/diastolic blood pressure, *HDL-C* high-density lipoprotein-cholesterol, *VAI* visceral adiposity index. *: if *P* < 0.05 between group 0 [AHI (0-5)] and group 1 [AHI(5-15)]; **: if *P* < 0.05 between group 0 [AHI (0-5)] and group 2 [AHI(15-30)]; ***: if *P* < 0.05 between group 0 [AHI (0-5)] and group 3[AHI(30-)]; ****: if *P* < 0.05 between group 1 and group 2; *****: if *P* < 0.05 between group 1 and group 3; ******: if *P* < 0.05 between group 2 and group 3

**Fig. 1:**
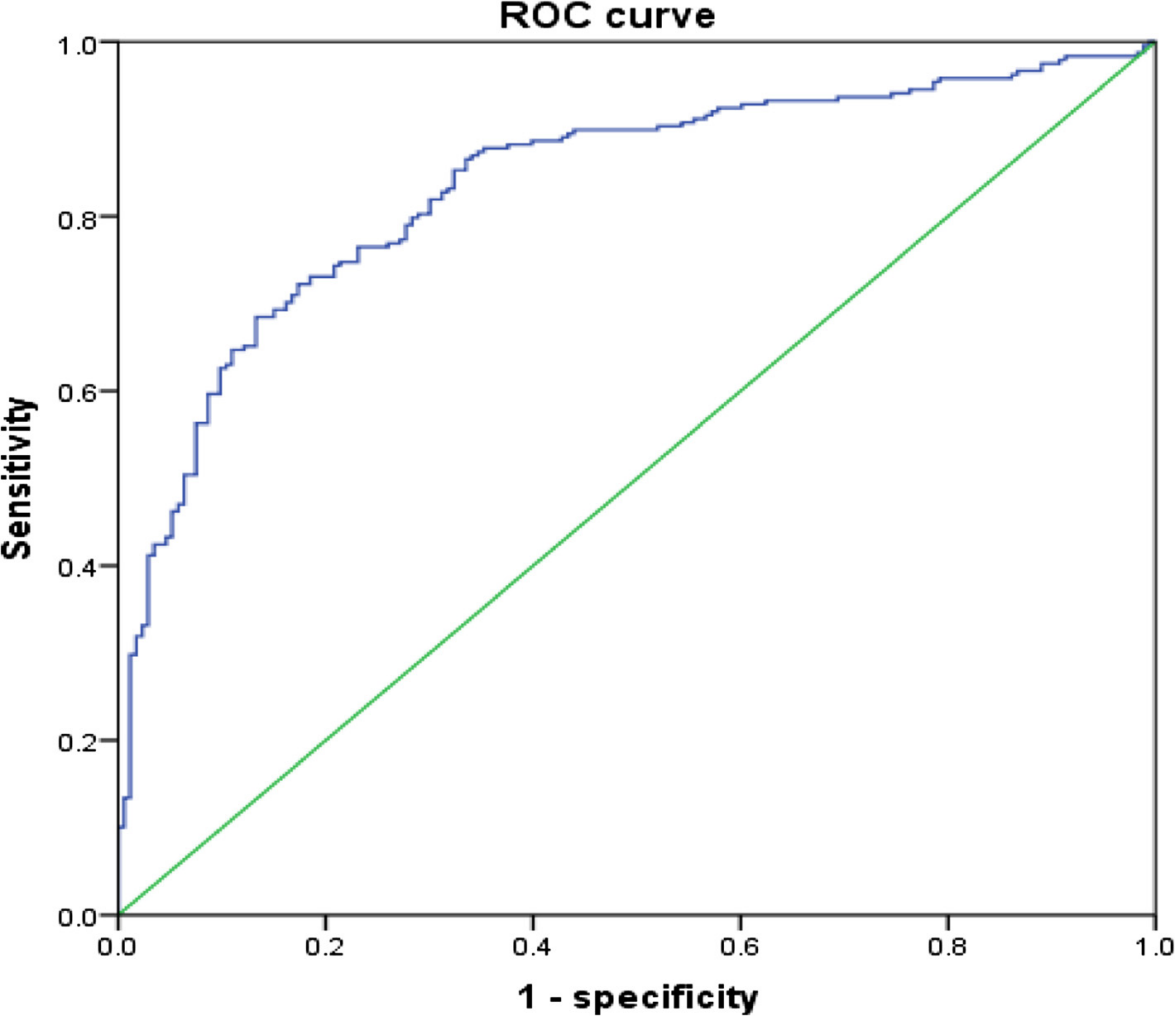
ROC curves for prediction of the presence of MetS in all subjects with suspected OSA. The area under ROC curves was observed for visceral adiposity index. (AUC = 0.836, 95 % CI: 0.797–0.875, *p* = 0.000). A VAI of 2.282 had a sensitivity of 68.5 % and a specificity of 86.7 % in determining the occurrence of MetS. ROC: Receiver operating characteristic; MetS: metabolic syndrome; OSA: obstructive sleep apnea; AUC: area under curve; CI: confidence interval. VAI: visceral adiposity index

**Fig. 2:**
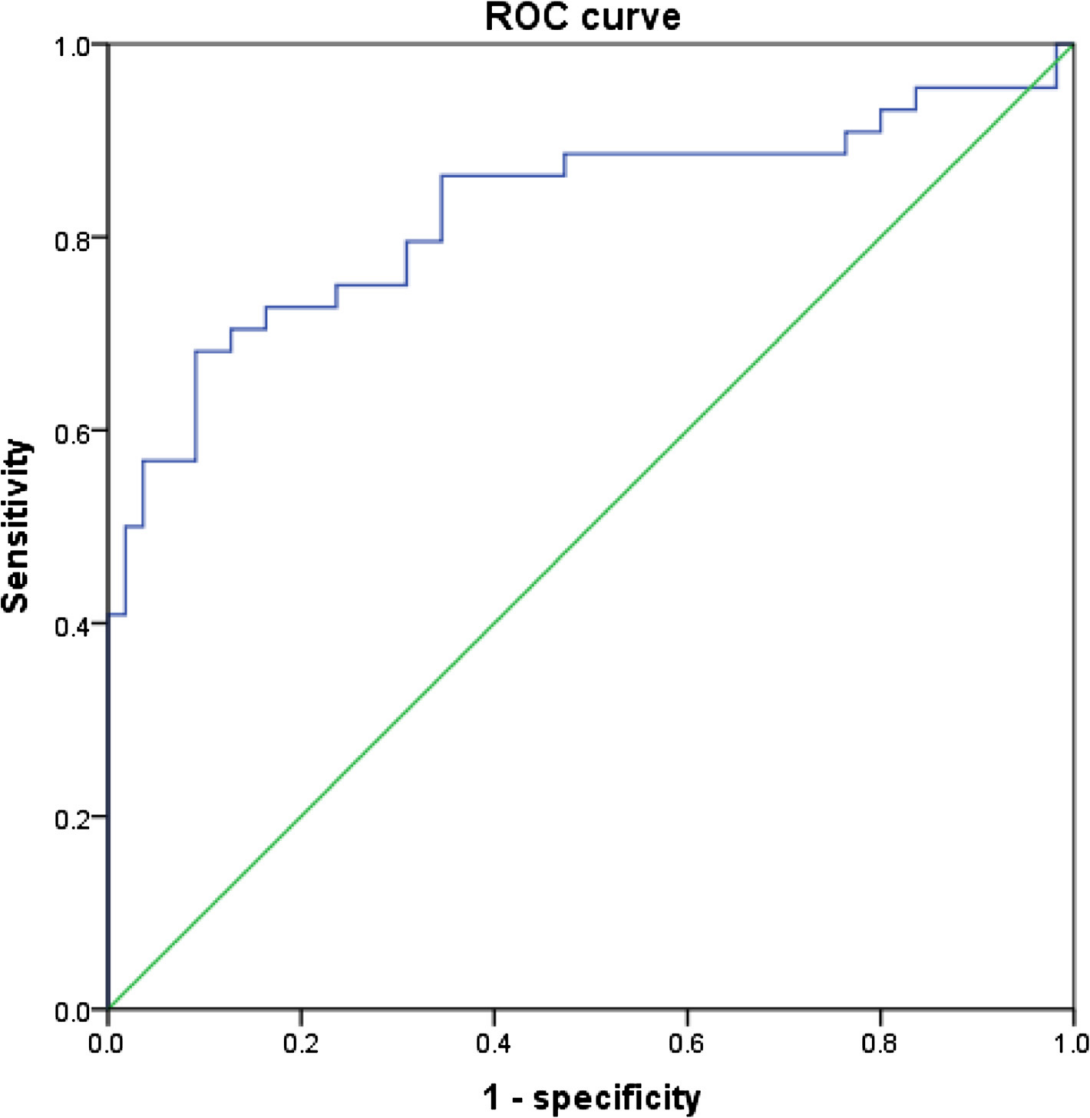
ROC curves for prediction of the presence of MetS in males with OSA. The area under ROC curves was observed for visceral adiposity index. (AUC = 0.838, 95 % CI: 0.792–0.883, *p* = 0.000). A VAI of 2.105 had a sensitivity of 73.3 % and a specificity of 82.1 % in determining the occurrence of MetS. ROC: Receiver operating characteristic; MetS: metabolic syndrome; OSA: obstructive sleep apnea; AUC: area under curve; CI: confidence interval. VAI: visceral adiposity index

**Fig. 3:**
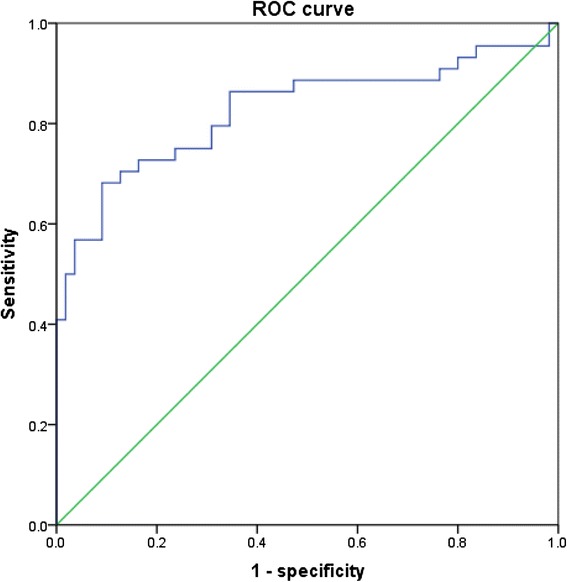
ROC curves for prediction of the presence of MetS in females with OSA. The area under ROC curves was observed for visceral adiposity index. (AUC = 0.826, 95 % CI: 0.736–0.916, *p* = 0.000). A VAI of 2.511 had a sensitivity of 68.2 % and a specificity of 90.9 % in determining the occurrence of MetS. ROC: Receiver operating characteristic; MetS: metabolic syndrome; OSA: obstructive sleep apnea; AUC: area under curve; CI: confidence interval. VAI: visceral adiposity index

Table 3 shows the correlations between VAI, OSA-related parameters and other metabolic variables. Irrespective of gender, VAI was all significantly correlated with WC, BMI, AHI, mean SpO_2_, LaSO_2_, HDL-C and UA (all *p* < 0.05). Unfortunately, the significant association of VAI with other indices did not existed concerning TS90 %, SBP, FG, TG in females and ODI, DBP in males. A VAI of 2.282, 2.105, 2.511(for all subjects, males and females) were calculated from ROC curves to determine the occurrence of MetS in OSA patients. The related sensitivity and specificity by sex are detailed in Figs. 1, 2 and 3. The area under the ROC curve of VAI was 0.836 (0.797–0.875), 0.838 (0.792–0.883), 0.826 (0.736–0.916) (all *p* < 0.001). According to the cut-offs, OSA patients were prone to get involved in significantly greater risk in incident MetS, with logistic regression after adjusting confounders such as age and current smoking (odds ratio [OR] = 10.237, *p* = 0.000; OR = 13.556, *p* = 0.000; OR = 21.458, *p* = 0.000).

**Table 3:** Spearman’s rank correlation coefficients between VAI and polysomnographic and metabolic characteristics

	Log VAI (All)		Log VAI (Male)		Log VAI (Female)	
	β	*p*	β	*p*	β	*p*
Log AHI	0.272	0.000	0.219	0.000	0.401	0.000
Log mean SpO_2_	−0.178	0.000	−0.160	0.005	−0.215	0.032
Log LaSO2	−0.165	0.001	−0.160	0.005	−0.272	0.007
Log TS90%	0.157	0.001	0.129	0.023	0.187	0.064
Log ODI	0.282	0.000	0.035	0.695	0.410	0.000
Log SBP	0.113	0.000	0.120	0.172	0.245	0.014
Log DBP	0.171	0.000	0.141	0.014	0.187	0.064
UA	0.305	0.000	0.261	0.000	0.368	0.000
Log FG	0.487	0.002	0.107	0.059	0.266	0.008
TG	0.897	0.000	0.897	0.000	0.930	0.000
Log HDL-C	−0.667	0.000	−0.660	0.000	−0.778	0.000
Log metabolic score	0.698	0.000	0.694	0.000	0.674	0.000

*VAI* visceral adiposity index, *AHI* apnea-hypopnea index, mean SpO2 mean nocturnal oxygen saturation, *LaSO2* lowest O2 saturation, TS90% the percentage of sleep duration with SpO2 < 90 %, *ODI* oxygen desaturation index, *SBP/DBP* systolic/diastolic blood pressure, *UA* uric acid, *FG* fasting glucose, *TG* triglycerides, *HDL-C* high-density lipoprotein-cholesterol

## Discussion

This cross-sectional study demonstrated that there were significant differences in terms of OSA-related indices, UA, incidence of MetS, metabolic score and VAI grouped by AHI. Spearman correlation illustrated that regardless of gender, VAI was all significantly correlated with PSG characteristics as AHI, mean SpO_2_, LaSO_2_ and metabolic indicators as UA. Particularly, the correlation of VAI with metabolic score existed. Further, all suspected OSA patients or by sex according to certain cut-offs of VAI were prone to get involved in MetS (OR = 10.237, *p* = 0.000; OR = 13.556, *p* = 0.000; OR = 21.458, *p* = 0.000, respectively).

It has been emphasized that there is a high co-prevalence rate of OSA and MetS [20, 21]. As elucidated, OSA was closely correlated with typical features of the MetS, i.e. IR and accumulation of visceral adipose tissue (VAT) [3, 8, 22]. Numerous studies in obese patients [2, 3, 8, 22] with OSA have shown that VA is significantly higher than BMI—matched controls. Indeed, excess VA was strongly associated with the presence and severity of apnea, especially in males [22]. Likewise, despite of similar BMI and WC, Vgontzas et al. revealed that LaSO_2_ during sleep was independently associated with VAT, which was in agreement with us expressed by VAI(Table 3) [3]. Further evidence was provided by LaSO_2_ which significantly predicted IR [23], supporting that LaSO_2_ may be one of the underlying promoters in inducing lipid abnormality [24]. On the other hand, as discussed by Shah et al. in 1,511 individuals, different impact of visceral fat assessment by CT on MetS risk existed. Irrespective of BMI, initial VA and change in VA were associated with MetS. Dynamic inspection in VA may be predictive in the target of therapy in cardio-metabolic diseases [2]. Collectively, visceral obesity/IR may be the principal determiners, progressively leading to the exacerbation of MetS and OSA. Meanwhile, progressive deterioration of OSA may accelerate worsening VA and MetS with nocturnal elevations of hormones, such as cortisol and insulin.

VAI, a simple indicator strongly correlated with VA, was obtained from several metabolic parameters. It is suggestive of a possible ‘adipose tissue dysfunction’ and the risk of IR, MetS and CVD in populations without an overt MetS [10, 11, 15, 16, 25]. The current data regarding whether VAI is a reliable parameter of OSA severity remain conflicting. Significances could be found in VAI among groups classified by AHI, irrespective of gender. Furthermore, significant correlations were displayed in VAI and OSA-related variables (Table 3). In contrast, gender-specific research inferred that VAI increased with IR linking with VA, and it was not predictive of OSA [13]. Mazzuca et al. suggested gender-related interactions between OSA, VA and metabolic abnormalities, unfortunately, VAI was not a good indicator of OSA Notably, VA increased with OSA severity, and the significant effects of ethnicity on VA have been extensively investigated. East Asians have the most deleterious abdominal fat distribution [9], particularly, in Asian women [26]. Excess VA and OSA are often related conditions, and they seem to interact with each other in a vicious circle. Although VAI was not a gold standard for VA, VAI may change in line with the increase in VA to some degree. This discrepancy may be attributed to differences in the races, the OSA criterion and higher BMI in their study [13]. As far as we were concerned, VAI reflected accurately the degree of VA and IR [13, 18, 27]. Further, in a cohort study [18] enrolling 308 adult Saudi subjects, metabolic, hormonal parameters and circulating concentrations of several circulating adipokines such as adiponectin, leptin were examined. VAI was finally identified as the sole determinant of adiponectin, one of the chief players in the MetS progress [28]. The fact that VAI is related only with adiponectin levels may account for the detrimental role of VA in the loss of defensive mechanisms and the occurrence of cardio-metabolic dysfunction. It should be emphasized that Chinese individuals are predisposed to have more VA than Westerners, which suggests an increased risk of metabolic abnormalities [9]. Compared with other metabolic biomarkers, VAI, which combined both physical and metabolic parameters, was better in identifying metabolism abnormalities and abdominal obesity instead of MRI and CT [10, 14]. Moreover, regardless of the ability of CT as the gold standard in discriminating fat distribution, it could not be easily applicable in population and clinical practice due to high cost and radiation [29]. Application of VAI may be of clinically importance and public health implications in the management of MetS.

Though we have shown that the metabolic score increased with the exacerbation of OSA, which was in line with many researchers [6, 20], interestingly, metabolic score and VAI had the strongest correlation with the polysomnographic indices of ODI in our study. It may remind us the key impact of oxyhemoglobin desaturations on the metabolism [30]. It was possible that oxyhemoglobin desaturations affected VAI more than apnea or hypopnea. Caution should be paid that focusing on nocturnal oxygen would be beneficial to the prevention of these diseases.

The impact of OSA on inflammatory markers in consecutive patients with MetS has been reported. Meanwhile, accumulation of VAT has been associated with an increase in interleukin-6, tumor necrosis factor-a, hs-CRP, and a decrease in adiponectin in obese patients [31]. Also, available information supported the positive correlation between TNF and VAI [32], implying a role of VAI in proinflammatory activity. Besides, the strongest association between VAT with hyperuricemia was clearly in favor of us (Table 3) [33]. Increased UA levels have been shown to play a mechanistic role in inflammation and antioxidation in the environment of obesity, which may in turn promote lipid oxidation [34]. The close relation between VAI and UA indicated that VAI could be also used to predict inflammation and metabolism, which was confirmed by the dose–response relationship of UA with MetS [35]. In addition, hyperinsulinemia would contribute to hyperuricemia and hypertension [36], resulting in an increased risk of metabolic abnormality.

Consistent with our findings (Table 3), the significant link of VAI with both prehypertension and hypertension was observed [37]. Despite of the absence of the BP factor in the calculation of VAI, it still could be utilized as an effective biomarker for monitoring hypertension. Previous studies have emphasized the clinical importance of the applicability of VAI in predicting MetS [14, 16]. Notably, they drew more attention to the correlation of VAI with some components of MetS [16]. By contrast, on the basis of OSA, we explored the indicative role of VAI in the occurrence of MetS. Early stages of MetS may be not highlighted by the classic criteria. However, VAI, seems to be able to indicate early metabolic risk in patients without overt MetS by suggesting continuous changes. Moreover, different from the fact that diagnosis of MetS should be in accordance with three or more components, VAI was obtained from an equation. Thus, a combination of VAI and NHANES-ATP III criteria could estimate metabolic abnormality in OSA patients complementarily. Further, it was important to point out that the occurrence of MetS was associated to the development of CVD and diabetes mellitus type 2 in teenagers and adults [38, 39]. As shown [38], the MetS was responsible for 2.6–3.0 times higher CVD mortality. Appropriate stratified-for-age cut-off point of VAI from a large cross-sectional study was able to identify a supposed adipose tissue dysfunction associated with CVD [15]. Similarly, we determined the risk of MetS according to VAI cut-offs by sex, especially in OSA patients. As displayed in Table 4, we implied that females would be disposed to greater risk of MetS than males, although excess VA was more strongly associated with apnea, especially in males. Early recognition, prevention of metabolism abnormalities in OSA patients would improve the management of these disorders.

**Table 4:** Odds ratio for MetS in OSA patients according to the cut-off of VAI

Factor	OR	95%CI	*p*
VAI (All)	10.237	5.842-19.939	0.000
VAI (Male)	13.556	7.563-24.298	0.000
VAI (Female)	21.458	6.940-66.345	0.000

According to the cut-off of VAI, OSA patients above the cut-offs tended to suffer from significantly greater risk in incident MetS, with logistic regression after adjusting confounders such as age, current smoking. R^2^ for entire model = 0.333, 0.367, 0.460 (for all subjects, males and females, respectively), *CI* confidence interval, *OR* odds ratio, *MetS* metabolic syndrome, *OSA* obstructive sleep apnea, *VAI* visceral adiposity index

Some limitations of our study still need to be elucidated. First, due to the cross-sectional design, we were unable to determine a direct causal relationship between OSA and metabolic abnormalities. Likewise, the statistical power of VAI to predict the MetS in OSA patients may be insufficient. Second, IR was not measured, therefore we could not directly examine the associations of VAI with IR which was strongly associated with VA and inflammation [40]. Third, we failed to consider the menopausal status, which may lead to some bias in final inferences. Increases in VAT were supposed to be associated with menopausal transition and the ages [41, 42], however, relatively little of the variance in VAT (or VAT%) was due to the independent effect of menopausal status [42]. Well designed studies, including strict exclusion for menopausal women, are needed to figure out the exact association between VAI, OSA and MetS. Forth, significances in the gender proportion among groups existed, which may affect some inferences after statistics analysis. However, we analyzed the role of VAI by sex, making a contribution to a comprehensive understanding on these disorders. Finally, differences from previous reports in geographical and ethnically diverse populations, in part, attenuated these concerns.

## Conclusion

Overall, VAI was significantly associated with MetS and OSA. As a simple and alternative approach obtained in everyday practice, VAI may offer a powerful tool to identify patients with OSA at risk of MetS.

## Abbreviations

OSA: obstructive sleep apnea
MetS: metabolic syndrome
ROC: receiver operating characteristic
CT: computed tomography
AHI: apnea–hypopnea index
mean SpO_2_: mean oxygen saturation
LaSO_2_: lowest oxygen saturation
TS 90 %: the percentage of sleep duration with SpO_2_ < 90 %
ODI: oxygen desaturation index
WC: waist circumference
BMI: body mass index
SBP: systolic blood pressure
DBP: diastolic blood pressure
HDL-C: high density lipoprotein-cholesterol
FG: fasting glucose
TG: triglycerides
VAI: visceral adiposity index
CVD: cardiovascular diseases
VA: visceral adiposity
VAT: visceral adipose tissue
IR: insulin resistance
PSG: polysomnography
